# Are Danish doctors comfortable teaching in English?

**DOI:** 10.1186/s13104-016-2229-6

**Published:** 2016-08-27

**Authors:** L. Nilas, E. C. Løkkegaard, J. B. Laursen, J. Kling, D. Cortes

**Affiliations:** 1 Department of Obstetrics and Gynecology 537, Hvidovre University Hospital, 2650 Hvidovre, Denmark; 2 Faculty of Health Science, University of Copenhagen, Copenhagen, Denmark; 3 Department of Obstetrics and Gynecology, Nord Zeeland University Hospital, Hilleroed, Denmark; 4 Centre for Internationalization and Parallel Language Use, University of Copenhagen, Copenhagen, Denmark; 5 Department of Pediatrics, Hvidovre University Hospital, Hvidovre, Denmark

**Keywords:** International students, Clinical teaching, Teaching in foreign language, Doctors’ English skills, Self-assessment

## Abstract

**Background:**

From 2012–2015, the Departments of Obstetrics and Gynecology and of Pediatrics at the University of Copenhagen conducted a project, “Internationalization at Home ”, offering clinical teaching in English. The project allowed international students to work with Danish speaking students in a clinical setting. Using semi-quantitative questionnaires to 89 clinicians about use of English and need for training, this paper considers if Danish clinical doctors are prepared to teach in English.

**Results:**

The majority self-assessed their English proficiency between seven and eight on a 10 unit visual analogue scale, with 10 equivalent to working in Danish, while 15 % rated five or less. However, one-fourth found teaching and writing in English to be twice as difficult than in Danish, and 12 % rated all teaching tasks in English at four or less compared to Danish. The self-assessed need for additional English skills was perceived low.

**Conclusion:**

Teaching in English was rated as 30 % more difficult than in Danish, and a significant subgroup of doctors had difficulties in all forms of communication in English, resulting in challenges when introducing international students in non-native English speaking medical departments.

## Background

Current strategic policy at the University of Copenhagen (UCPH) aims to internationally promote education, create partnerships and collaborate in high level scientific study [1]. One specific area of internationalization is the inclusion of non-Danish students in traditionally Danish degree programs. For example, in 2013 the Faculty of Health at UCPH enrolled 116 international students, both full time students and Erasmus exchange students [2, 3]. To meet the needs of this more diverse student body, courses traditionally taught in Danish were taught in English.

At present, the majority of English medium instruction (EMI) clinical courses at the School of Medical Sciences (MedSchool) are taught through traditional lectures and elective clinical training sessions. Since 2006, UCPH has also offered a limited number of clinical courses in hospital departments taught in English. In contrast to classroom based EMI medical courses, these clinical training courses offer local and international students the opportunity to study and work together in a hospital setting with local patients and offer the Danish students to interact with the international students.

While enrolment of international students in courses at UCPH’s MedSchool has resulted in challenges related to both linguistic and cultural diversity for the students, it has also affected the teaching staff, and lecturers are adjusting to this change. Whereas some of the clinical doctors have been positive to teaching in English, there has also been some resistance to internationalization of courses and the use of English as the language of instruction and teaching. To investigate possible causes and determine whether any of these are related to linguistic proficiency, we asked Danish doctors in this clinical setting to consider how comfortable they are working and teaching in English.

## Methods

### Setting

In 2012, a 3-year educational project entitled Internationalization at Home, was introduced at the departments of Obstetrics and Gynecology (ObGyn) and Pediatrics (Ped) at Hvidovre Hospital and Nordsjaellands Hospital in Hilleroed. Each 5-week course concluded with an oral examination. Of the 6–10 students in each course, half were local UCPH students, who voluntarily registered for this English medium programme, while the other half came from various European countries.

The language of instruction of these courses was English, not only during formal lectures and case-based learning session, but was also intended at departmental meetings. The morning meetings, where acute referrals, plans for patient care, and teaching are discussed, were scheduled to be conducted in English when the international teams consisting of both the Danish and the international students were present. One of the departments chose to keep the daily morning meetings in Danish, but instead offer more intensive special English-spoken morning meetings for the international student team.

### Survey

Using a paper-based questionnaire, distributed at morning meetings at three of the departments, we asked the doctors to reflect on their English proficiency, including frequency of language use and level of difficulty communicating in English in the workplace, as compared to Danish usage.

Information about the frequency with which the doctors listen to, read, write, speak and lecture in English was collected using a semi-quantitative questionnaire with five response options: never, rarely, sometimes, often, and very often. To further investigate whether attitudes about English proficiency were related to age and teaching experience, the questionnaire included questions about age, experience and gender, but not charge. On a continuous 10 cm visual analogue scale (VAS) with questions related to specific tasks, we asked the doctors to state the ease by which they read, understand, write, guide, teach, and possibly conduct examinations in English compared to in Danish, where 0 represents very difficult and 10 unproblematic and as easy as in Danish. For comparison, information was also collected on how the doctor’s perceived these tasks in Danish. Furthermore, doctors were asked to self-assess their overall proficiency in English from very poor to expert level. Finally, the doctors were requested to assess their need for additional English language skills for the future on a scale ranging from currently satisfied to in great need for improved proficiency.

### Statistical analyses

Of 91 questionnaires distributed during a morning meeting, two were only partly completed leaving 89 for analyses. Responses on the 10 cm VAS were read by 0.5 cm accuracy. A response of having performed an examination once was interpreted as having examination experience. For the questions comparing the ease of performing in English compared to Danish, the values on the VAS were interpreted in percentage differences i.e., a VAS of seven for teaching is interpreted as 30 percent more difficult than in Danish. Numeric outcomes are given as geographical means (means calculated on log transformed values), ranges, and 25–75 percentiles. Statistical differences were tested by Student’s-*t* test using a level of significance of 5 %.

### Ethics

Informed consent was not formally asked for, but the respondents were anonymous and they could decline participation. No formal ethics approval was required for our study according to Danish law.

## Results

### Use of English by the respondents

**Fig. 1:**
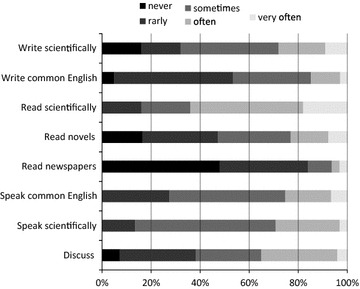
How often do you read, write, speak, and participate in discussions in English?

**Fig. 2:**
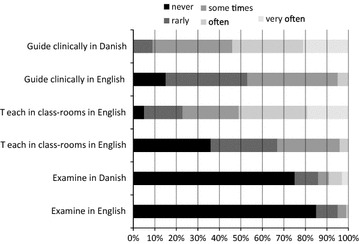
How often do you guide clinically, teach in classrooms, and conduct examinations in Danish and English?

More than 60 % of the respondents indicate they often or very often read English medical texts; the corresponding figures for reading fiction and newspapers were 20 and 5 % respectively (Fig. 1). Written English was mostly used in an academic context, and less than one-third of the respondents often speak English. About 40 % rarely or never participate in discussions in English. The majority of the respondents had been involved in clinical bedside teaching of medical students, mostly in Danish (82 %, 74/89), whereas half (49 %, 44/89) have experienced bed-side teaching of English speaking students (Fig. 2). Only a minority of the respondents had experience in performing lectures or examinations in English.

### Self-assessment of English skills

**Fig. 3:**
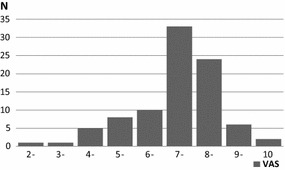
Self-assessed English skills: How do assess your overall proficiency in English? VAS ranging from very poor (0) to expert-level (10)

The majority of respondents reported a self-assessment of between seven and eight on a VAS scale for general English performance, but 17 % rated their competencies at five or less (Fig. 3). The general skills in English showed a declining trend with age with average values of 7.3, 7.2, 6.7 and 6.0 for the age groups 29–39, 40–49, 50–59 and 60+ years, respectively. Those who examined occasionally or frequently (n = 17) were older than the 72 without experience in examinations (50.2 versus 42.8 years, p < 0.05), but there was no difference between the two groups self-assessment of their general, professional or daily English language skills. There was no difference between the overall English skills in obstetricians/gynecologists and pediatricians, nor between men and women.

Regarding the more specific tasks, the respondents generally rated their skills for reading English high, but about 30 % more difficult than in Danish (Table 1). The respondents reported that they were comfortable with writing professionally, lecturing, classroom teaching and guiding clinically in Danish, with mean values of 8.6, 8.6, 8.9 and 9.2, respectively, and 25–75 percentiles between eight and ten. The mean values for performing these tasks in English compared to in Danish were 6.4, 6.4, 6.6 and 6.5, respectively, with 25 percentiles around five (Table 1), indicating that one in four respondents found teaching and writing in English to be twice as difficult as in Danish. Eleven (12 %) of the respondents rated all teaching tasks in English at four or less compared to Danish, indicating great difficulties in all forms of communication in English.

**Table 1:** Self-assessed language proficiency

	English compared to Danish		Danish	
	Mean	Median (range), 25–75 percentiles	Mean	Median (range), 25–75 percentiles
Read medical literature	7.7	7.5 (0.2–10), 6.8–8.5		
Read novels	7.2	8 (3–10), 7–10		
Read English newspapers	6.7	7 (1.5–10), 6–9		
Understand spoken daily language	7.9	8 (3.0–10), 7–10		
Understand news programs	7.7	8 (2.0–10), 7–9		
Write professionally	7.5	8 (3–10), 8–9	8.6	9 (2–9), 8–10
Lecture students/colleagues	6.4	7 (0.5–10), 5.5–8.5	8.6	9 (2–10), 8–10
Guide clinically	6.4	7 (0.5–10), 5–9	9.2	9 (7–10), 9–10
Teach in classrooms	6.6	7 (1.0–10), 6–8	8.9	9 (6–10), 8–10
Conduct examinations (n = 17)	6.5	7 (0–10), 5–8		

How easy is it for you to read, write and teach in English compared to in Danish? VAS ranging from 0, very difficult, to 10, which is just as easy as in Danish

n = 89. Data are given as geometric means, range and 25–75 percentiles

**Fig. 4:**
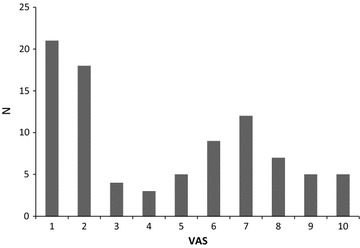
Self-assessed need for further linguistic English competencies. VAS ranging from no need (0) to large need (10)

The self-assessed need for further skills in English varied from a no need (0) to a great need (10), but the majority had little interest in improving their linguistic skills (Fig. 4). The need of additional language skills was not different between those with teaching and examination duties and those without.

## Discussion

The study shows that teaching and communication in English compared to Danish is assessed to be about 30 % more difficult, and that approximately 10 % of doctors find it very difficult to teach and communicate orally in English.

English is the primary foreign language in Denmark and is taught for at least 7 years during primary school. Some of the medical text books at the MedSchool are in English, but English has not previously been used as the language of instruction. Specialist doctors are to some degree trained to acquire medical knowledge in English, but an academic career demands ability also to communicate in English, as 90 % of the medical scientific literature is disseminated in English. The participating departments are common clinical departments where approximately half of doctors are specialists and half are younger doctors at different stages of specialized training. Some doctors have been on the wards for many years and others have just joined the team. Two of the departments have had a few English speaking students for several years, while teaching in English had only just been introduced at the third department at the time of the study. With 30–52 new students per semester at each department, it is necessary to draw on the skills of all doctors at the departments, as confirmed by the fact that over 80 % of the respondents, regardless of level of medical responsibility, stated that they work with students in the clinical setting. The daily classroom lessons are shared between lecturers/professors, but may also be led by senior trainees/residents, as training in teaching is an essential part of the specialist medical training program, a common trend at university hospitals [4–6]. The introduction of EMI courses was a pragmatic decision. The doctors received no formal supervision or offer of English language training. Those who had difficulties communicating in English could to some extent avoid formal teaching, but they were still faced with meetings held in English and interaction with students on the ward.

The questionnaire used in this study was not validated and relies on the physicians’ self-assessment, and generally self-assessment has been found to correlate poorly with performance in some contexts [5]. Doctors generally rate themselves highly as teachers [4], and often assume they have teaching qualifications without courses in medical pedagogy [6, 7]. It is also recognized that teachers often overestimate their linguistic abilities, their teaching and their communicative skills [8]. However, as we asked about the clinician’s assessment of the ease of teaching and instructing in English compared to in Danish, we assume that any overestimation may be equivalent in the two situations. The survey was performed during morning meetings and the respondents were expected to be representative for the staff. We have no comparable data from other departments, but find no reason to believe that the doctors in our departments differ substantially from other clinicians in a hospital setting.

This study reports the doctors’ self-assessed perceptions of strong receptive English language skills, i.e., listening and reading comprehension. Several of the doctors stated that they have fair competences in writing medical English, but while many found it difficult, they often also found writing medical Danish challenging. However, overall, the respondents in this study found oral production in teaching in English to be the most challenging, indicating that receptive language skills not were sufficient to ensure productive competences. This supports previous research, which suggests that the teachers find themselves less confident when teaching in English compared to their own Scandinavian language [9, 10]. In addition, previous studies have reported that doctors’ EMI teaching was more formal and contained less small-talk compared to teaching in Danish [9–11].

The self-assessed need for further English skills varied. Those who found their linguistic abilities sufficient ranged from one mother-tongue English speaker, to others who had lived in English speaking counties either as children or had studied abroad. In addition, there were some who may have felt their English knowledge was sufficient, because they were on their way toward careers as general practitioners.

Introducing education in a foreign language is associated with many challenges [10, 11], and in a clinical setting the challenges are not only linguistic, but also involve attitudes and opinions, and demand structural changes in the organization, and are time consuming. Danish Universities demand internationalization and acknowledge that linguistic competences for both medical students and teachers need to improve. UCPH has established a center for support, the Centre for Internationalisation and Parallel Language Use (CIP), which develops courses for improving the English skills for university staff [12–14]. A study from CIP [14] reported, that the attitudes of the academic staff towards increased use of English as the educational language at the University of Copenhagen, depended on the teaching load in English. While the MedSchool is increasingly offering courses held in English, it is doubtful if such initiatives can ameliorate the linguistic shortcomings in a clinical department, as a clinical course also involves communication with patients and other groups of employees, who may find English communication even more difficult. We find that teaching English-speaking students is a challenge to a clinical department, and our results question whether internationalization through teaching in English in clinical departments is the best possible model.

## Abbreviations

EMI: english-medium instruction
IaH: Internationalisation at Home
MedSchool: School of Medical Sciences (MedSchool)
UCPH: University of Copenhagen
VAS: visual analogue scale

## References

[1] University of Copenhagen, Strategy 2016. http://research.ku.dk/strategy/. Retrieved 25 April 2016.

[2] University of Copenhagen, Studieophold i udlandet. http://udrejse.ku.dk/Udveksling_Feltarbejde_og_Praktik/Europa/english/. Retrieved 22 Feb 2015.

[3] University of Copenhagen, Statistikberedskab. http://tal.ku.dk/boks1/Statistikberedskab_2013__Studienoegletal.pdf. Retrieved 25 April 2016.

[4] Busari J, Scherpbier AJJA, van der Vleuten CPM, Essed GGM (2003). The perceptions of attending doctors of the role of residents as teachers of undergraduate students. Med Educ..

[5] Eva KW, Regehr G (2011). Exploring the divergence between self-assessment and self-monitoring. Adv Health Sci. Educ..

[6] Erie JA, Starkman SJ, Pawlina W, Lachman N (2013). Developing medical students as teachers: an anatomy-based student-as-teacher program with emphasis on core teaching competencies. Anat Sci Educ.

[7] Butani L, Paterniti DA, Tancredi DJ, Li S-T (2013). Attributes of residents as teachers and role models—a mixed methods study of stakeholders. Med Teach.

[8] Hellekjaer GO, Westergaard MR. An exploratory survey of content learning through English at Scandinavian universities. In: Simensen AM editor. Teaching and learning foreign languages. Issues and ideas. Oslo: Unipub; 2002. p. 47–61.

[9] Tange H (2010). Caught in the tower of Babel: University lecturer’s experiences with internationalisation. Lang Intercult Commun..

[10] Hellekjær GO, Wilkin R, Zegars V (2007). The implementation of undergraduate level English medium programs in Norway: An explorative case study. Researching content and language integration in higher education.

[11] Borgan M, Kase BF, Bjerkeset S, Handal G. Engelskspråklig undervisning i medisinutdanningen. En evaluering af prøveordning med engelsk som undervisningsspråk i 9. semester. Oppdragsgruppen ved Pedagogisk Forskningsinstututt. Universitetet i Oslo, det Utdannensvitenskapelige Fakultet. 2005.

[12] Internationalisation and language competencies at the University of Copenhagen. Centre for Internationalisation and Parallel Language Use. http://cip.ku.dk/satsningsomraader/pulje2016internationalisering/. Retrieved 25 April 2016.

[13] Gregersen F (Ed). 2014. Hvor parallelt. Om parallellspåkighet på Nordens universitet. http://rafhladan.is/bitstream/handle/10802/6165/TN2014535%20web.pdf?sequence=1. Retrieved 25 April 2016.

[14] Attitudes towards English as the language of instruction among Danish university lecturers at the University of Copenhagen. Centre for Internationalisation and Parallel Language Use. University of Copenhagen. 2009. http://cip.ku.dk/pdf/publ/Underviseres_holdninger_til_engelsk_som_undervisningssprog.pdf. Retrieved 23 April 2016.

